# Apologies Repair Trust via Perceived Trustworthiness and Negative Emotions

**DOI:** 10.3389/fpsyg.2019.00758

**Published:** 2019-04-03

**Authors:** Fengling Ma, Breanne E. Wylie, Xianming Luo, Zhenfen He, Rong Jiang, Yuling Zhang, Fen Xu, Angela D. Evans

**Affiliations:** ^1^Department of Psychology, Zhejiang Sci-Tech University, Hangzhou, China; ^2^Department of Psychology, Brock University, St. Catharines, ON, Canada; ^3^Zhejiang Technical Institute of Economics, Hangzhou, China

**Keywords:** apology, trust, perceived trustworthiness, negative emotions, transgression

## Abstract

The present study examined whether perceptions of a transgressor’s trustworthiness mediates the relationship between apologies and repaired trust, and the moderating role of negative emotions within this process. Chinese undergraduate students (*N* = 221) completed a trust game where they invested tokens in their counterpart, and either experienced no trust violation (i.e., half of the tokens returned), a trust violation (i.e., no tokens returned), or a trust violation followed by an apology. Participant’s trust behavior was measured by the number of tokens they re-invested in their counterpart in a second round of the game. Participants also completed measures to assess perceptions of the transgressor’s trustworthiness and emotional state. Results revealed that participants who received an apology were more likely to trust in their counterpart, compared to those who did not receive an apology, and this relationship was mediated by perceptions of the transgressor’s trustworthiness. Further, the relationship between apologies and perceptions of the transgressors trustworthiness was moderated by negative emotions; apologies only improved perceptions of trustworthiness for participants who experienced less negative emotions.

## Introduction

Trust is a fundamental component of social relationships, and it is the prerequisite for smooth and efficient interactions (Lewicki and Bunker, 1996; Balliet and Van Lange, 2013). Yet, trust is fragile and easily threatened or broken (Kramer, 1999; Kim et al., 2013; Schilke et al., 2013). Given the simultaneous importance and fragility of trust, researchers have identified strategies that can be utilized to repair trust after violations. For example, apologizing after a trust violation is often the first and one of the most commonly used techniques to repair damaged trust (Schweitzer et al., 2006; Fischbacher and Utikal, 2013; Walfisch et al., 2013). While existing studies have primarily focused on the effectiveness of an apology (e.g., Ferrin et al., 2007), little is known about “how” and “for whom” an apology can effectively rebuild trust following a violation. Therefore, the current study seeks to examine the role of emotions, and perceptions of the transgressor’s trustworthiness, as influential factors in the process of repairing trust.

To date, there is an extensive literature that suggests apologies are effective in repairing trust (Ferrin et al., 2007; Kim et al., 2006, 2009, 2013; Risen and Gilovich, 2007; Schniter et al., 2013; Walfisch et al., 2013). Even superfluous apologies, such as “I’m sorry about the rain!,” in which the apologizer is clearly not responsible for the event, have been found to increase perceived interpersonal trust (Brooks et al., 2014). Further, studies utilizing the economic trust game have found that following a trust violation, adults who receive an apology are more likely to trust in their partner in the future, compared to adults who did not receive an apology (e.g., Schniter et al., 2013). Taken together, these findings highlight the positive influence of an apology in repairing trust.

Past research, however, has only begun to examine *how* apologies repair trust (e.g., Schniter and Sheremeta, 2014). One possible explanation for this effectiveness may be the perceptions an individual has about the trustworthiness of the transgressor following an apology. For example, when confronted with violations that lead to damaging trust (e.g., betrayal, transgressions, cheating, unfairness), an individuals’ perceptions of the transgressor may be damaged. Past research suggests that the act of an apology can reduce these negative perceptions of the transgressor (Darby and Schlenker, 1982, 1989) and signal intentions and propensity for future trustworthiness (Schniter and Sheremeta, 2014). Although research suggests that apologies improve perceptions of the transgressor’s trustworthiness, and can repair trust, no study has examined these perceptions as the underlying mechanism for successfully repairing trust. Thus, the present investigation aims to assess whether the perceived trustworthiness of the transgressor mediates the relationship between apology and post-violation trust, whereby an apology improves perceptions of the transgressor’s trustworthiness, and in turn increases trust behavior.

Importantly, repairing trust is a dynamic process of human interaction. Whether and to what extent an apology repairs damaged trust is not only influenced by transgressor’s efforts (e.g., offering an apology), but is also directly affected by the victim’s individual characteristics. Existing research has found wide individual variation during the process of trust repair (Schweitzer et al., 2006; Haselhuhn et al., 2010). For example, Haselhuhn et al. (2010) reported that implicit beliefs of moral character could moderate trust recovery. Another factor that may influence *for whom* an apology effectively repairs trust is the individuals’ negative emotional state triggered by the violator’s transgression. Existing studies have emphasized that emotions influence subsequent game behavior, and more specifically the damaging role of negative emotions (Fehr and Gächter, 2002; Dunn and Schweitzer, 2005; Hopfensitz and Reuben, 2009; Kausel and Connolly, 2014). For example, Dunn and Schweitzer (2005) found that positive emotions induced by irrelevant settings increase one’s intention to trust others, and induced negative emotions (e.g., anger) decrease such trust. Further, Schniter and Sheremeta (2014) found that positive emotions experienced following an apology mediated the relationship between apologies and trust repair, such that receiving an apology improved positive emotions, which in turn increased trust behavior. While it is clear that emotions influence an individual’s propensity to trust, no prior work has considered how emotions triggered by a trust violation might influence the effectiveness of a subsequent apology in repairing trust. It is possible that an individual who experiences extreme negative emotions (e.g., betrayal, devastation), may be less receptive to an apology, compared to an individual who finds a violation of trust to be a minor inconvenience. Therefore, the present investigation also seeks to examine whether negative emotions moderate the relationship between apologies and trust repair.

The current study is the first to directly assess “how” and “for whom” apologies repair trust. That is, past research has largely focused on the benefits of giving an apology (e.g., politeness, saving face; Goffman, 1967), as well as the direct relationship between receiving an apology and trust repair (e.g., Schniter et al., 2013). However, the current study uniquely examines factors that contribute to the effectiveness of apologies in repairing the receiver’s trust (i.e., perceived trustworthiness and emotions). Undergraduate students from a university in China completed a two-round trust game (based on Berg et al., 1995). In the first round of the trust game, participants invested tokens in their counterpart. The partner’s decision was pre-determined, so that the counterpart demonstrated fair and reciprocal behavior (Control condition), violated the partner’s trust (No-Apology condition), or violated the partner’s trust followed by offering an apology (Apology condition). The second round of the trust game, where participants were given the option to re-invest tokens in their counterpart, served as a measure of post-violation trust behavior. Participants also completed measures of their perceptions of the counterpart’s trustworthiness, as well as their self-reported emotional state following the trust violation.

**FIGURE 1:**
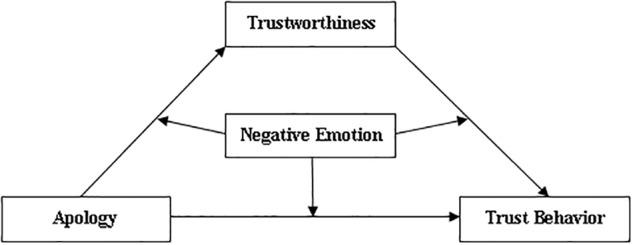
The hypothetical moderated mediation model.

Given that participants in the control condition did not experience a trust violation, they were expected to pass significantly more tokens to their counterparts, compared to participants in Apology and No-Apology conditions where trust violation occurred. However, following a trust violation, it was predicted that participants would pass significantly more tokens after receiving an apology compared to when no apology was received. Given that apologies have been found to improve perceptions of the transgressor and act as an indicator for trustworthiness (Darby and Schlenker, 1982, 1989; Schniter and Sheremeta, 2014), it was expected that an apology would increase perceptions of the counterparts’ trustworthiness, and lead to more trusting behavior in the future. That is, perceptions of trustworthiness were expected to mediate the relationship between apologies and trust. Furthermore, we assessed the moderating role of negative emotions on the relationship between apologies and trust. A moderated mediation analysis (see Figure 1 for hypothesized model) was performed to examine both the direct (apologies repair trust) and indirect (apologies repair trust via perceptions of the counterparts’ trustworthiness) pathways between apology and trust behavior. Overall, it was expected that an apology would be less effective in repairing trust for individuals who experienced more negative emotional reactions to the transgression.

Within the context of a trust paradigm, it is possible that gender also plays a role in participants’ behaviors. Past studies have found that men are more likely to invest tokens in their counterpart compared to woman, despite the risk that their partner may violate their trust and fail to return any of the proceeds (Snijders and Keren, 1999; Chaudhuri and Gangadharan, 2002; Buchan et al., 2008). It is suggested that men exhibit more trusting behaviors (e.g., send more money) because they are more optimistic about what they will receive in return (Buchan et al., 2008). Therefore, the present study will examine whether gender influences trust behavior and perceptions of the counterparts trustworthiness. If gender differences are found, gender will be controlled for in the moderated mediation analyses to assess whether emotions and perceived trustworthiness play a role in the relationship between apologies and trust, above and beyond gender.

## Materials and Methods

### Participants

Two hundred and thirty-eight undergraduates from a university in Zhejiang, China (student population of approximately 27,000 students) participated in this study. Twelve participants were excluded for failing to invest their tokens on the first trial and five participants were excluded for expressing suspicions that the counterpart was not a real person. Thus, the final sample included 221 participants (44.3% male, *M_age_* = 19.45, *SD* = 1.05) who were randomly assigned to one of three conditions: Apology (*n* = 62, 46.8% male, *M_age_* = 19.62, *SD* = 1.46), No Apology (*n* = 87, 41.4% male, *M_age_* = 19.30, *SD* = 1.22), or Control (*n* = 72, 45.8% male, *M_age_* = 19.76, *SD* = 1.15). This study was carried out in accordance with the recommendations of the Institutional Review Board of the Zhejiang Sci-Tech University with written informed consent from all subjects. All subjects gave written informed consent in accordance with the Declaration of Helsinki. The protocol was approved by the Institutional Review Board of the Zhejiang Sci-Tech University.

### Design and Procedure

Upon arrival to the laboratory, all participants were settled into individual testing rooms. Participants were informed that they would be completing a decision-making exercise and that the amount of money they received for participating in the study would depend on the decisions they made during the task. Participants were told that they would be playing a trust game with an anonymous counterpart (matched on sex and age) in the next room (Berg et al., 1995). Unbeknownst to participants, there was no actual counterpart in the next room and participants received pre-set responses based on their assigned condition.

In the first round, the trustworthiness of the counterpart was established. Participants were given 20 tokens, and told that they could either pass all of the tokens to their counterpart or keep all of the tokens. If they passed the 20 tokens to their counterpart the money would be tripled to 60 tokens., and their counterpart would then decide how many tokens to return to the participant (0–60 tokens). Participants were instructed to indicate their decision on a piece of paper and place it in an envelope. If participants chose to keep the 20 tokens, the test procedure was terminated, and they received 10 RMB for their time. If participants chose to pass the 20 tokens to the counterpart, the experimenter would take the envelope into the next room and wait 2 min before returning with the counterpart’s decision. Upon their return the experimenter passed the envelop to the participant and said, “Here is your partner’s decision; you can open it to see how many tokens s/he returned to you.” (translated from Mandarin).

The decision made by the counterpart was predetermined based on the condition randomly assigned to the participant. Participants in the Control condition received 30 tokens in return (half of the counterpart’s awarded amount), representing trustworthy behavior. Participants in the two trust violation conditions (Apology and No Apology conditions) received 0 tokens in return, representing untrustworthy behavior. Participants in the Apology condition then received a note from their counterpart stating, “I’m really sorry I didn’t return 30 tokens to you, it’s not fair to you and I take responsibility.” (translated from Mandarin).

Directly following the first round of the trust game, participant’s trusting behavior was assessed through a second round of the trust game. Participants again were given 20 tokens that they could either pass to their counterpart or keep. However, in this round, they were not required to pass the full amount. Participants could decide how many of the 20 tokens to pass to their counterparts. If they chose to keep the tokens, they could exchange the tokens for a corresponding cash reward. If they chose to the pass tokens to their counterpart, the tokens would again triple in value, and the counterpart would decide how many tokens (0–60) to return. After participants recorded their decision and placed it in the envelope, the experimenter then told participants that they would take the envelope to the counterpart and would return with the counterpart’s decision. While participants were waiting for the counterpart’s decision, they completed a measure of perceived trustworthiness to the counterpart and a negative emotion evaluation scale. Once all scales were complete, participants were interviewed by the experimenter about any potential suspicions about the paradigm and were fully debriefed. All participants received 10 RMB for their time.

### Measures

#### Trust Behavior

Participants trust behavior was measured by the number of tokens passed to their counterpart in the second round of the trust game. Higher scores indicated more trusting behavior (range 0–20).

#### Trustworthiness

As a measure of participants’ perception of counterpart’s trustworthiness, participants were asked to indicate on a 5-point scale (1 = strongly disagree, 5 = strongly agree) how strongly they agreed with the statement, ‘I believe that the partner will make a fair distribution in the next round.’ (translated from Mandarin).

#### Negative Emotions

Participants also completed an emotions questionnaire (based on Tsang, 2007; Carlisle et al., 2014). Participants were asked to rate four possible negative emotions (angry, resentful, hurt and fearful, α = 0.83) based on how they were currently feeling toward their partner’s behavior on a 5-point scale (responses range from 1 = *strongly disagree* to 5 = *strongly agree*). For example, “I feel anger toward him/her,” “I feel hurt by him/her.” Negative emotion scores were calculated by averaging the four items.

## Results

### Trusting Behavior

**FIGURE 2:**
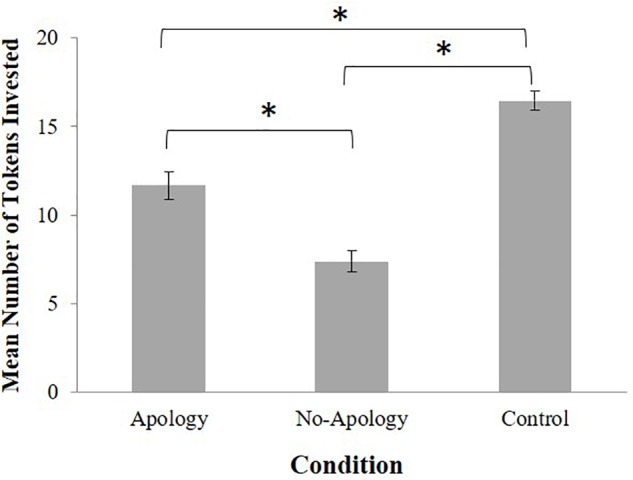
Mean number of tokens invested in partner (trusting behavior) by condition where more tokens represent more trusting behavior. Error bars represent standard errors. ^∗^*p* < 0.001.

To assess the effects of a trust-violation and apology on participants’ trusting behaviors a 3 (Condition: Apology, No-Apology, Control) × 2 (Gender: male, female) between-participants factorial analysis of variance (ANOVA) was performed on participants’ trust behavior scores. There was a significant main effect of condition, *F*(2, 215) = 53.49, *p* < 0.001, η*_p_^2^* = 0.33. Consistent with our predictions, *Post hoc* comparisons [least significant difference (LSD)] indicated that participants passed the greatest number of tokens in the Control condition (no trust violation; *M* = 16.44, *SD* = 4.60), followed by the Apology condition (*M* = 11.66, *SD* = 6.23), and the least amount of tokens in the No-Apology condition (*M* = 7.38, *SD* = 5.49) (between Control and Apology condition: *p* < 0.001; between Control and No-Apology condition: *p* < 0.001; between Apology and No-Apology condition: *p* < 0.001) (see Figure 2). A significant main effect of gender was also found, *F*(1,215) = 11.81, *p* = 0.001, η*_p_^2^* = 0.05, with males passing significantly more tokens to their counterpart (*M* = 13.22, *SD* = 6.49) compared to females (*M* = 10.73, *SD* = 6.52). No significant interaction was found, *F*(2,215) = 0.59, *p* = 0.557, η*_p_^2^* = 0.005.

### Trustworthiness

**FIGURE 3:**
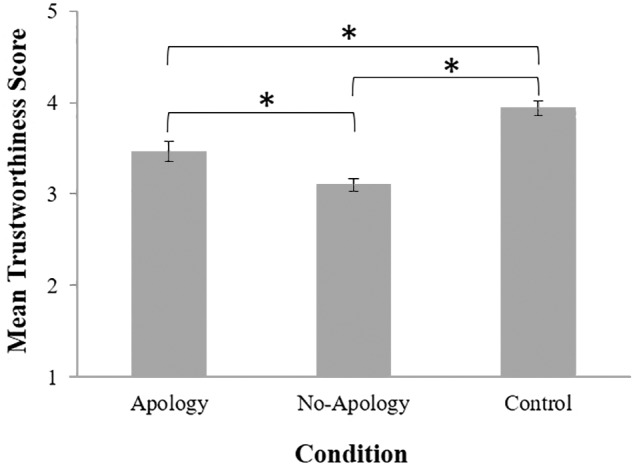
Mean evaluation of trustworthiness scores by condition where higher scores represent more trustworthiness. Error bars represent standard errors. ^∗^*p* < 0.001.

To assess whether the trust violations as well as the apology influenced participants’ perceptions of the counterpart’s trustworthiness, a 3 (Condition: Apology, No-Apology, Control) × 2 (Gender: male, female) between-participants factorial ANOVA was performed on participants trustworthiness scores. There was a significant main effect of condition, *F*(2, 215) = 24.38, *p* < 0.001, η*_p_^2^* = 0.19. Consistent with our predictions, *Post hoc* comparisons (LSD) indicated that participants rated their counterpart to have significantly higher trustworthiness in the Control condition (*M* = 4.12, *SD* = 0.58), followed by the Apology condition (*M* = 3.56, *SD* = 0.77), and the lowest trustworthiness in the No-Apology condition (*M* = 3.13, *SD* = 0.79) (between Control and Apology condition: *p* < 0.001; between Control and No-Apology condition: *p* < 0.001; between Apology and No-Apology condition: *p* = 0.003) (see Figure 3). A significant main effect of gender was also found, *F*(1,215) = 5.11, *p* = 0.025, η*_p_^2^* = 0.02, suggesting that males (*M* = 3.62, *SD* = 0.75) perceived their counterpart to have greater trustworthiness, compared to females (*M* = 3.40, *SD* = 0.83). There was no significant interaction, *F*(2,215) = 1.25, *p* = 0.289, η*_p_^2^* = 0.011.

### Negative Emotions

**FIGURE 4:**
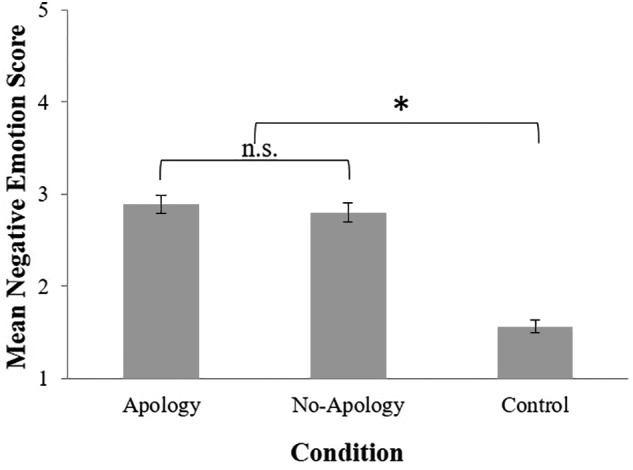
Mean negative emotion scores by condition where higher scores represent more negative emotions. Error bars represent standard errors. ^∗^*p* < 0.001.

Next, we examined the effects of a trust-violation and apology on participants’ self-reported negative emotions. A 3 (Condition: Apology, No-Apology, Control) × 2 (Gender: male, female) between-participants factorial ANOVA was performed on participants’ negative emotion scores. There was a significant main effect of condition, *F*(2, 215) = 64.93, *p* < 0.001, η*_p_^2^* = 0.38. *Post hoc* comparisons (LSD) indicated that participants experienced less negative emotions in the Control condition (*M* = 1.56, *SD* = 0.56), compared to participants in the Apology (*M* = 2.89, *SD* = 0.81) and No-Apology conditions (*M* = 2.80, *SD* = 0.91), *p*s < 0.001. There was no significant difference between the Apology and No-Apology condition, *p* = 0.643 (see Figure 4), suggesting that participants negative emotions were influenced by the trust violation, regardless of whether or not an apology was given. There was no significant main effect of gender, *F*(1,215) = 1.28, *p* = 0.260, η*_p_^2^* = 0.006, and no significant interaction, *F*(2,215) = 1.61, *p* = 0.202, η*_p_^2^* = 0.015.

### The Mediation and Moderation Effects Analyses

Structural Equation Modeling (SEM) was used to assess the relationship between apologies, evaluations of trustworthiness, negative emotions, and trusting behavior. The model was analyzed in Mplus v. 7 (Kline, 2011) using maximum likelihood estimation. A moderated mediation model was used to further understand factors that influence the relationship between apologies and trust. We used the following indices to assess fit: comparative fit index (CFI), where values <0.95 indicate good fit; Tucker-Louis index (TLI), where values >0.90 indicate good fit; and root mean square error of approximation (RMSEA), where values <0.05 are a good model fit.

### The Mediating Effect of Trustworthiness in the Association Between Apology and Trust Behavior

First, we assessed whether perceptions of the partner’s trustworthiness mediated the relationship between apologies and trust, for participants who experienced a trust violation. Given that participants in the Control condition did not experience a trust violation, they were excluded from further analyses.

Preceding the mediation analysis, we examined partial correlations between apology, trusting behavior, and perceptions of trustworthiness, controlling for gender, for the Apology and No-Apology conditions where trust violations occurred. Results revealed that all variables were significantly related to each other, which met the requirements for completing a mediation analysis (see Table 1).

**Table 1:** The partial correlations for apology, trustworthiness, and trust behavior (controlling for gender).

Variables	1	2	3
1. Apology^a^	–		
2. Trustworthiness	0.22^∗∗^	–	
3. Trust behavior	0.34^∗∗^	0.53^∗∗^	–

*^a^Apology was dummy coded such that 0 = without an apology and 1 = with an apology. ^∗∗^p < 0.001.*

**FIGURE 5:**
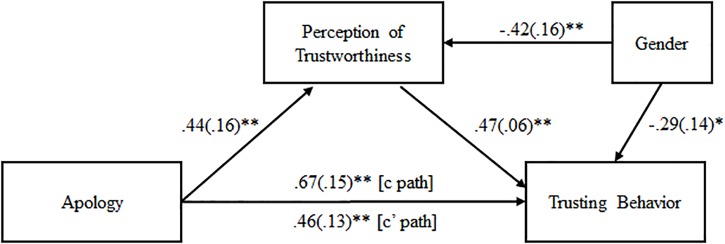
Mediation model of the relationship between apology and trust with perception of trustworthiness as the mediator. Each pathway includes unstandardized path coefficients of the direct relationship of one variable to another, adjusted for gender. The *c* path represents the total effect of apology on trusting behavior. The *c’* path represents the direct effect of apology on trusting behavior after accounting for evaluations of trustworthiness. Standard errors are shown in parentheses. ^∗^*p* < 0.05; ^∗∗^*p* ≤ 0.001.

A mediation analysis was performed with apology as the predictor, perceptions of trustworthiness as the mediator, and trust behavior as the outcome variable. Gender was included as a covariate. The mediation model was fully saturated *X*^2^/*df* = 0.00, CFI = 1.00, TLI = 1.00, RMSEA = 0.00). All path coefficients among apology, perceptions of trustworthiness, and trust behavior were significant (see Figure 5). The total effect of apology on trusting behavior was significant (*b* = 0.67, *SE* = 0.15, *t* = 4.40, *p* < 0.001). The direct effect of apology on trusting behavior, controlling for trustworthiness, was reduced though remained significant (*b* = 0.46, *SE* = 0.13, *t* = 3.62, *p* < 0.001). However, a Sobel test was conducted and found partial mediation in the model (*z* = 2.595, *p* = 0.009).

### The Moderating Effect of Negative Emotion on the Direct Association Between Apology and Trust Behavior

Next, we assessed whether negative emotions moderated the relationship between apologies and trusting behavior, controlling for gender (see Figure 1 for predicted model). A moderation analysis was performed with apology as the predictor, negative emotions as the moderator, and trust behavior as the outcome variable. The model was fully saturated (*X*^2^/*df* = 0.00, CFI = 1.00, TLI = 1.00, RMSEA = 0.00). Both the main effects of apology and negative emotions were significant unique predictors of trust behavior (see Table 2). However, there was no significant interaction between apology and negative emotions, suggesting that negative emotions did not moderate the relationship between apologies and trust behavior.

**Table 2:** The moderating effect of negative emotions on trust behavior.

Predictor	*b*	*SE*	*t*	*p*
Apology	0.71	0.14	4.91	<0.001
Negative Emotion	−0.31	0.09	−3.55	<0.001
Apology X Negative Emotion	−0.14	0.13	−1.06	0.290

### The Moderated Mediation Effect of Trustworthiness and Negative Emotion on the Association Between Apology and Trust Behavior

**FIGURE 6:**
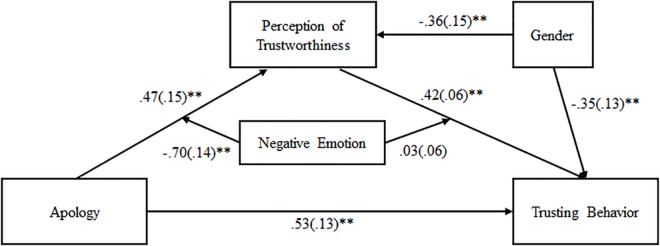
Moderated mediation model of the relationship between apology and trust behavior, with negative emotions as the moderator, and perception of trustworthiness as the mediator. Each pathway includes unstandardized path coefficients of the direct relationship of one variable to another, adjusted for gender. Standard errors are shown in parentheses. ^∗^*p* < 0.05; ^∗∗^*p* ≤ 0.001.

To further assess the relationship between apology and trust behavior, we used a moderated mediation analysis to examine whether negative emotions moderated the relationship between apologies, trustworthiness, and trust behavior. The model was a good fit (*X*^2^/*df* = 1.34 < 2, CFI = 0.99, TLI = 0.97, RMSEA = 0.048). Negative emotion moderated the relationship between apology and trustworthiness (path a), *b* = −0.70, *SE* = 0.14, *t* = −4.94, *p* < 0.001, but did not moderate the relationship between trustworthiness and trust behavior (path b), *b* = 0.03, *SE* = 0.06, *t* = 0.41, *p* = 0.680 (see Figure 6). Analysis of the moderation effect for apology and trustworthiness was conducted using simple slope tests calculated at −1 *SD* and +1 *SD* from the mean of negative emotion. Results indicated that participants who received an apology were more likely to perceive their partner as trustworthy if they experienced less negative emotions (*b* = 1.17, *SE* = 0.19, *t* = 6.13, *p <* 0.001), though this relationship between apology and perceptions of trustworthiness was not significant for participant’s who experience more negative emotions (*b* = −0.22, *SE* = 0.22, *t* = −1.03, *p* = 0.300).

## Discussion

The present study examined the effectiveness of an apology in repairing broken trust, as well as how and for whom this relationship occurs. Consistent with our predictions, we found that an apology can effectively improve perceptions of the transgressor’s trustworthiness as well as trusting behaviors. Importantly, we also found that the effects of an apology on trust were partially mediated by perceptions of the transgressor’s trustworthiness, and negative emotions moderate the relationship between apologies and perceptions of the transgressor trustworthiness.

Consistent with previous findings, our results indicated that following a transgression, individuals who received an apology passed a greater number of tokens (representing more trusting behavior), compared to those who did not receive an apology (Kim et al., 2006, 2009, 2013; Ferrin et al., 2007; Risen and Gilovich, 2007; Schniter et al., 2013; Walfisch et al., 2013). These findings suggest that an apology can indeed improve trust behavior. Similarly, apologies were able to repair perceptions of the individuals’ trustworthiness. These results replicated and provide support for previous findings that have shown how apologies can alleviate damage to trust evaluations following a violation (e.g., Ferrin et al., 2007). Taken together this study suggests that apologies influence both behavioral (e.g., investment of tokens) and cognitive (i.e., perceptions of trustworthiness) propensities to trust. Notably, while apologies improved trust, trust was not fully restored. This is consistent with past research that suggests an apology cannot rebuild trust to the level it was before the violation occurred. For example, Schniter and Sheremeta (2014) found that participants who experienced a transgression yet received an apology demonstrated lower trust in their partner, compared to participants who had never experienced a trust violation. Therefore, it is possible that alternative repairing strategies (e.g., denial, promises, compensation) in addition to apologies may more effectively repair trust. Future studies should seek to examine whether apologies in comparison, as well as in combination with other repairing strategies (e.g., denial, promises, compensation) can successfully repair trust.

Our findings also revealed gender differences when assessing both cognitive and behavioral trust. That is, males were more likely to view their counterpart as trustworthy, and demonstrated more trusting behaviors, compared to females. This is consistent with past research that suggests males are more likely to demonstrate trust in relationships (Snijders and Keren, 1999; Buchan et al., 2008), perhaps due to greater expectations for reciprocation. While our findings replicate previous research, we were further interested in *how* and *for whom* apologies repair trust, above and beyond previously established gender differences.

Past literature has begun to examine processes by which apologies effectively repair trust (Schniter and Sheremeta, 2014), yet no study has examined perceptions of trustworthiness as the mechanism for this relationship. Results revealed that perceptions of trustworthiness partially mediated the relationship between apologies and trust, where an apology improved perceptions of the transgressor’s trustworthiness, which in turn increased trusting behavior. This contributes to the current literature suggesting that trust is in part a cognitive process (e.g., Lewis and Weigert, 1985; McAllister, 1995). Importantly, trust only partially mediated this relationship. Therefore, future studies should take other factors (e.g., social norms) into consideration to explain *how* apologies repair trust. Future studies should also consider alternative methodologies for obtaining perceptions of trustworthiness. In the current study, perceptions of trustworthiness are obtained through self-report, however strategies such as physiological (e.g., heart rate, skin conductance) and neurological assessments might better capture participants perceptions of a transgressors trustworthiness.

While the process of repairing trust largely depends on the actions of the transgressor (e.g., demonstrating remorse through an apology), this process also depends on the individual who experienced the transgression. It is known from past studies that emotions influence an individual’s propensity to trust (e.g., Dunn and Schweitzer, 2005; Schniter and Sheremeta, 2014). However, the current study is the first to examine the degree to which an individual is emotionally affected by a trust violation, and whether this influences the effectiveness of a subsequent apology. When examining the direct relationship between apologies and trust, negative emotions were not a significant moderator. This suggests that regardless of how negatively an individual felt following a transgression, an apology effectively repaired trust. However, when examining the indirect association between apologies and trust, results revealed that negative emotions moderated the relationship between apologies and perceptions of the transgressor’s trustworthiness. That is, for individuals who experience more extreme negative emotions, apologies did not improve perceptions of trustworthiness for the transgressor. Alternatively, for individuals who experienced less negative emotions, apologies did improve perceptions of the transgressor’s trustworthiness. These findings suggest that for individuals who are severely impacted by the trust violation, a simple and basic apology alone, as offered in the current trust paradigm, cannot repair trust perceptions. Future studies should seek to examine whether alternative strategies may be effective in repairing trust for individuals who demonstrate a strong negative emotional reaction to trust violations. Further, given that the current study used a between-subjects design, it is possible that other individual differences, beyond emotions, influence trust repair. As such, future studies should examine the influence of individual differences (e.g., trust tendencies) on the relationship between apologies and trust.

It is important to note that in the current study, the emotions and trust perceptions scales were administered after participants made their decision on how many tokens to re-invest in their counterpart following the transgression. It is possible that the act of re-investing tokens improved participants emotions and perceptions of their partner because they had shown forgiveness. Therefore, while these scales asked participants to reflect on their emotional state and perceptions of the transgressor following the trust violation, their current positive emotional state after re-investing tokens may influence their memory of how negatively they felt following the transgression. Importantly the retrospective nature of these assessments allow us to assess the relationship, but not the causal direction between apologies and emotions as well as trust perceptions. Future studies should experimentally examine participants emotions and trust perceptions directly following the trust violation and after an act of forgiveness to further examine the causal effect of an apology on emotional states and perceptions of a transgressor’s trustworthiness.

It is also interesting to note that emotions did not moderate the relationship between trust perceptions and trust behavior. It is possible that once perceptions of the transgressor’s trustworthiness are formed, it is those perceptions, rather than the individuals’ emotions, that influence their behavior. Future studies should seek to examine other factors that may moderate the relationship between trust perceptions and trust behavior, to understand for whom cognitive trust (i.e., perceptions) translates into trust behavior (i.e., re-investing tokens following a trust violation).

While apologies have often been examined in communication literature as a means for the transgressor to save face (Goffman, 1967), they can also largely benefit the receiver of the apology. That is, apologies can repair the receiver’s trust, and willingness to trust the transgressor in the future. Importantly, the current study uniquely reveals that perceptions of the transgressor’s trustworthiness may be an underlying mechanism for the relationship between apologies and trust behavior. The current study is also the first to reveal that negative emotions moderate the effectiveness of apologies in repairing perceptions of the transgressor’s trustworthiness. These findings offer insight into the underlying processes involved in trust evaluation and trust repair. Further, importantly, these findings also shed light on the impact of an apology in real-world contexts such as online interactions. Specifically, our findings suggest that a written apology (without a face-to-face interaction) can repair trust. Given that much of our communication now occurs electronically (e.g., via e-mail or social media), our findings suggest that apologies may be effective in repairing trust in these online forms. This is critical given that violations of trust are common occurrences within various contexts in everyday life including both online and face-to-face. Altogether, understanding *how* and *for whom* apologies repair trust provides insight into factors that influence the development and maintenance of trusting relationships.”

## Author Contributions

FX conceptualized the study. FX, FM, and ZH designed the study. ZH, RJ, and YZ collected the data. FM and XL analyzed and interpreted the results. BW and FM wrote the manuscript. AE revised the article critically for important intellectual content. All the authors read and approved the submitted version.

## Conflict of Interest Statement

The authors declare that the research was conducted in the absence of any commercial or financial relationships that could be construed as a potential conflict of interest.
